# Methylmalonyl Coenzyme A (CoA) Epimerase Deficiency, an Ultra-Rare Cause of Isolated Methylmalonic Aciduria With Predominant Neurological Features

**DOI:** 10.7759/cureus.48017

**Published:** 2023-10-31

**Authors:** Rui Diogo, Inês B Rua, Sara Ferreira, Célia Nogueira, Cristina Pereira, Joana Rosmaninho-Salgado, Luísa Diogo

**Affiliations:** 1 Pediatrics, Reference Centre of Hereditary Metabolic Diseases, member of MetabERN; Centre for Child Development, Coimbra Hospital and University Centre, Coimbra, PRT; 2 Genetics, National Institute of Health Dr Ricardo Jorge, Porto, PRT; 3 Pediatrics, Reference Centre of Refractory Epilepsies, member of EpiCARE; Coimbra Hospital and University Centre, Coimbra, PRT; 4 Genetics, Coimbra Hospital and University Centre, Coimbra, PRT; 5 Pediatrics and Inherited Metabolic Diseases, Reference Centre of Hereditary Metabolic Diseases member of MetabERN; Centre for Child Development, Coimbra Hospital and University Centre, Coimbra, PRT

**Keywords:** intellectual disability, seizures, spasticity, methylmalonic aciduria, methylmalonyl-coa epimerase

## Abstract

Methylmalonyl coenzyme A (CoA) epimerase (MCE) converts D-methylmalonyl-CoA into L-methylmalonyl CoA in the final common degradation pathway of valine, isoleucine, methionine, threonine, odd-chain fatty acids, and cholesterol side chains. Methylmalonyl-CoA epimerase deficiency is an ultra-rare autosomal recessive disorder where methylmalonic acid, methylcitrate, 3-hydroxypropionate, and propionylcarnitine are accumulated. We describe two novel pediatric patients and review the previously reported cases of MCE deficiency.

Including our two novel patients, at least 24 cases of MCE deficiency have been described, with a broad clinical spectrum ranging from asymptomatic to severely neurologically impaired patients. Our patients are siblings of Arabic origin who presented with metabolic decompensation with coma and epilepsy during infancy. Methylmalonic aciduria was disclosed, L-methylmalonyl-CoA mutase deficiency was assumed, and they were treated accordingly. When first seen in our country, aged 10 and four years, respectively, both presented severe intellectual disability and spasticity. The younger had an ataxic gait, and the older was wheelchair-ridden. The study of the *MMUT*, *MMAA*, *MMAB*, and *MMADHC *genes was normal. Subsequently, the pathogenic variant c.139C>T (p.Arg47*) in the* MCEE* gene was identified in homozygosity in both patients, leading to the diagnosis of MCE deficiency (Online Mendelian Inheritance in Man (OMIM^®^) 251120, McKusick-Nathans Institute of Genetic Medicine, Johns Hopkins University, MD, USA). Most patients were homozygous for that variant (83% of the alleles). Correct diagnosis allowed treatment adequacy and genetic counseling.

Methylmalonyl-CoA epimerase deficiency shares a similar biochemical profile with other rare genetic disorders. Patients present with overlapping clinical features with predominant neurological manifestations; genetic testing is indispensable for diagnosis. We found no association between genotype and biochemical and clinical phenotypes.

## Introduction

Isolated methylmalonic aciduria (MMAuria) encompasses a group of autosomal recessive propionate degradation disorders resulting in methylmalonic acid (MMA) accumulation in body fluids. Propionate is derived from the catabolism of four amino acids (valine, isoleucine, methionine, and threonine), the odd-chain fatty acids, the side chain of cholesterol, and the metabolism of some species of gut bacteria [1]. Through the action of propionyl coenzyme A (CoA) carboxylase (PPCA), propionate’s derivative propionyl-CoA is converted into D-methylmalonyl-CoA, which is then isomerized into L-methylmalonyl-CoA by methylmalonyl-CoA epimerase (MCE). The L-methylmalonyl-CoA is converted into succinyl-CoA by methylmalonyl-CoA mutase (MMUT), requiring adenosylcobalamin (AdoCbl), one of the active forms of vitamin B12, as a co-factor. Subsequently, succinyl-CoA enters the Krebs cycle to generate energy. Isolated MMAuria is usually caused by MMUT deficiency (Online Mendelian Inheritance in Man (OMIM®) 251120; McKusick-Nathans Institute of Genetic Medicine, Johns Hopkins University, MD, USA). Less frequent disorders of AdoCbl metabolism (complementation groups cobalmin (cbl)A, cblB, and cbl-D variant 2; OMIM 251100, 251000, and 277410, respectively) and MCE deficiency (OMIM 251120) should be considered in the differential diagnosis [2].

The gene coding for MCE is *MCEE* and is located on chromosome 2p13.3 [3]. Biallelic mutations in this gene responsible for MCE deficiency have been reported in at least 22 patients [4-14]. The most common pathogenic variant, c.139C>T (p.Arg47*), is a nonsense variant, which has been identified in homozygosity in at least 17 patients [4-8,10,11,14] and compound heterozygosity with another *MCEE* variant in at least two patients [9,12]. The remaining three MCE cases have three different *MCEE* variants in homozygosity [6,11,13].

Common biochemical findings in MCE deficiency are raised urinary excretion of MMA, methylcitrate (MCA), 3-hydroxypropionate (3-HP), and elevation of blood propionylcarnitine (C3), with normal vitamin B12 and homocysteine levels [15]. Methylmalonyl-CoA epimerase deficiency has a broad clinical spectrum. While some cases are asymptomatic, others have a neurodevelopmental delay or intellectual disability, neurodegeneration, acute metabolic decompensations with metabolic acidosis, hyperammonaemia, and encephalopathy [1]. Here, we describe two new pediatric cases of MCE deficiency with severe intellectual disability, presenting with acute metabolic decompensation and neurological impairment. We reviewed the cases published in the English literature for a better characterization of this ultra-rare condition.

## Case presentation

Case report of patient 1

Patient 1 is a male born at term from consanguineous parents of Arabic origin. At one month of age, he presented with seizures and a coma during a febrile infection. Since then, he has been taking antiseizure medications. Two more coma episodes with seizures associated with febrile infections occurred in the first year of life. Subsequently, he developed severe failure to thrive and developmental delays. At five years of age, high blood levels of C3 and C3:C0 and organic aciduria with increased MMA, MCA, and 3-HP prompted the diagnosis of MMAuria, so he was treated based on the diagnosis of MMUT deficiency.

His family moved to Portugal when he was 10 years old. At this age, he presented severe intellectual disability, no verbal language, and very poor social interaction, with Vineland’s adaptative behavior of six months old. He was wheelchair-ridden, with axial hypotonia and limb spasticity. His gross motor function classification system (GMFS) was level 5 (transport in a manual wheelchair). He had feeding difficulties and was malnourished. He was on a protein-restricted diet (~0.7 g/kg/day), with a total energy value (TEV) of ~42 kcal/kg/day, medicated with oral cobalamin, carnitine, metronidazole, and valproic acid. Protein intake and TEV were increased to ~1.5 g/kg/day and ~80 kcal/kg/day, respectively, which allowed 25% weight gain in two months. The investigation was consistent with isolated MMAuria (Table 1) with an elevation of urinary MMA, serum MMA, blood C3, and plasma alanine, normal serum levels of vitamin B12, homocysteine, and prolactin, and low creatinine (43 μmol/L, r.v. 50-70).

**Table 1:** Patient 1's laboratory findings during follow-up MMA: Methylmalonyl acid; MCA: Methylcitrate; C3: Propionylcarnitine

Parameters	At age 11	At age 12	At age 13	Reference range
Serum MMA (µmol/L)	1.38	1.64	3.01	< 0.40
Urinary MMA (µmol/mmol Cr)	27.5	37	2.94	0-3.3
Urinary MCA (µmol/mmol Cr)	6.6	7.9	-	0.2-5.8
Blood C3 (µmol/L)	5.09	5.86	-	0.69-3.39
Plasma alanine (µmol/L)	1050	580	-	10-400

A genetic panel for inherited metabolic diseases (172 genes, including *MMUT*, *MMAA*, *MMAB*, and *MMADHC*; see Appendix A), performed by Next Generation Sequencing (NGS), using MiSeqTM (Illumina Inc., San Diego, CA, USA) and capture method SureSelectXT HS (Agilent Technologies, Santa Clara, CA, USA), was inconclusive. Subsequently, clinical exome sequencing was performed by NGS, and the variant c.139C>T (p.Arg47*) was identified in homozygosity, which confirmed the diagnosis of MMAuria due to MCE deficiency (OMIM 251120).

**Figure 1:**
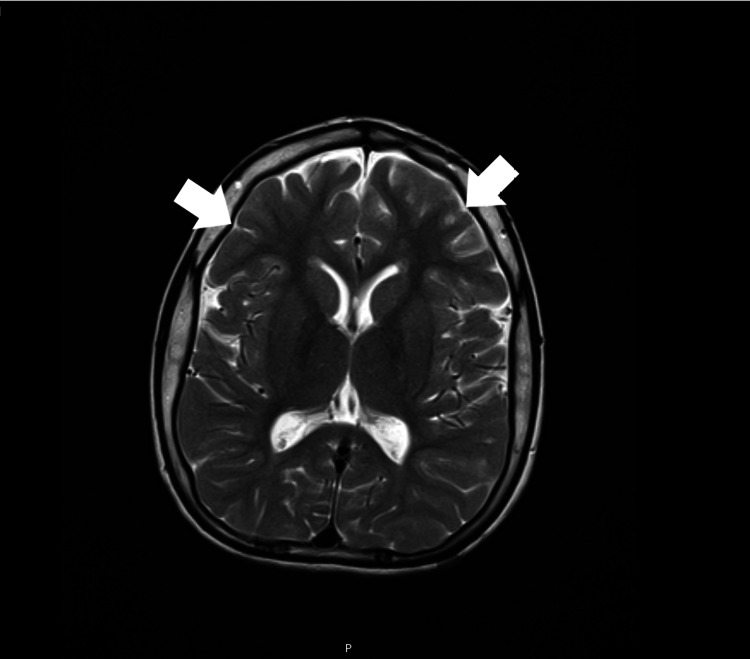
Patient 1's brain MRI, performed at the age of 13. Seen are diffuse bilateral cortico-subcortical moderate atrophy, mainly symmetrical; slight frontotemporal predominance (arrows); and thickening of the cranial vault.

The patient's urine organic acid profile showed intermittent mild excretion of MMA and MCA, and blood samples showed persistent elevation of serum MMA (1.08-3.01 μmol/L). The supplementation with vitamin B12 was stopped. At the age of 11, the EEG showed an encephalopathic background with multifocal paroxysmal activity. Brain MRI at age 13 showed diffuse cortical and subcortical atrophy, with frontotemporal predominance (Figure 1) and normal spectroscopy. Currently, at 13 years of age, under a protein-restricted diet (~1 g/kg/day), metronidazole, and carnitine, his weight is -3.6 SD, his length is -3 SD, and his head circumference is -2.2 SD. Epilepsy is controlled with valproic acid. He maintains axial hypotonia, limb spasticity, and severe intellectual disability with complete dependency on activities of daily living.

Case report of patient 2

Patient 2 is patient 1’s younger brother. Their parents and three older sisters are healthy. Apart from the brother, there are no other MMAuria or neurodegenerative diseases in the family. Patient 2 was born at term and presented with a coma and seizures during a febrile infection in the neonatal period. Investigation of this first episode led to the diagnosis of MMAuria at one month of age. He has been medicated for epilepsy ever since. Two metabolic decompensation episodes with a coma during febrile infections occurred in the first year of life. Hyperammonemia was reported in one of those episodes. At 13 months of age, the blood acylcarnitine profile disclosed an elevation of C3, and the urinary organic acid profile showed increased values of MMA, MCA, 3-HP, 3-hydroxyisovalerate, and 3-hydroxybutyrate. Epilepsy was controlled with phenobarbital, and severe neurodevelopmental delays arose. He was submitted to surgery for bilateral strabismus at four years of age.

He was five years old when his family moved to Portugal. At this age, significant neurodevelopmental delay with no verbal language and psychomotor agitation was evidenced by Vineland’s adaptative behavior at 9 months old. He presented with moderately generalized spasticity and a spastic gait. His GMFS was level 2 (walks with limitations). He had low weight (-2.3 SD), short stature (-2.7 SD), and microcephaly (-3.2 SD) on a protein-restricted diet (~0.9 g/kg/day) and a total TEV of ~51 kcal/kg/day. He was medicated with oral vitamin B12, carnitine, metronidazole, and phenobarbital. Protein intake and TEV were increased to ~1.5 g/kg/day and ~80 kcal/kg/day, respectively. Investigation revealed elevated urine MMA, serum MMA, and plasma C3 (Table 2) and normal plasma amino acid, homocysteine, vitamin B12, prolactin, and creatinine levels.

**Table 2:** Patient 2's laboratory findings at 13 months and from the age of five. MMA: Methylmalonyl acid; MCA: Methylcitrate; 3-HP: 3-hydroxypropionate; C3: Propionylcarnitine

Parameters	At 13 months	At age 5	At age 6	At age 7	Reference range
Serum MMA (µmol/L)	-	5.5	15.4	100.9	< 0.40
Urinary MMA (µmol/mmol Cr)	568.3	-	15.2	1230.9	0-3.3
Urinary MCA (µmol/mmol Cr)	227.0	-	-	-	0.2-5.8
Urinary 3-HP (µmol/mmol Cr)	166.0	-	-	-	0.6-21.5
Blood C3 (µmol/L)	12.07	4.43	-	-	0.69-3.39

**Figure 2:**
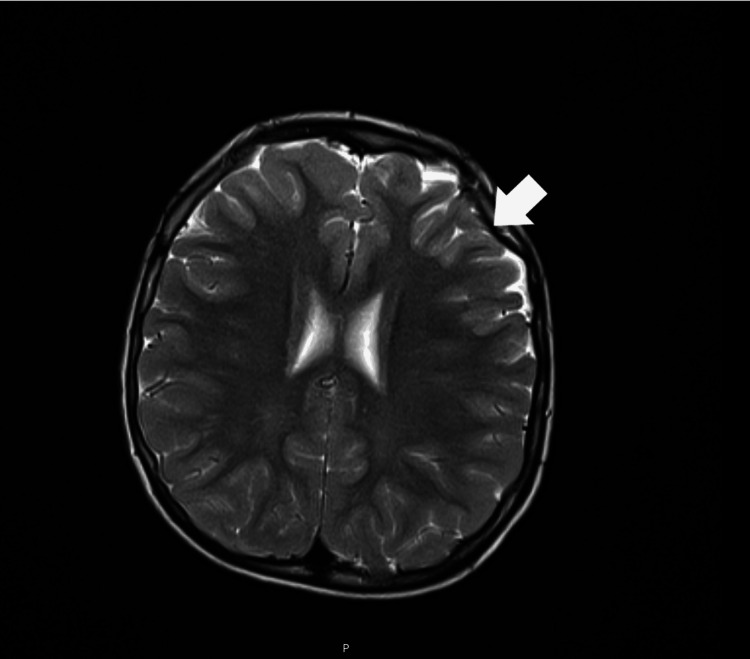
Patient 2's brain MRI, performed at the age of seven. Seen is a slight frontal bilateral atrophy, more evident on the left (arrow).

Sanger sequencing of exon 2 of the *MCEE* gene in patient 2 found the same homozygous variant c.139C>T (p.Arg47*) as his brother, confirming the diagnosis of MCE deficiency. Vitamin B12 was stopped. At age five, the EEG showed an encephalopathic background with paroxysmal occipital activity with left predominance. Brain MRI at age seven showed a subtle frontal atrophy with left predominance (Figure 2). Brain spectroscopy was normal. Currently, at the age of seven, he has a severe intellectual disability and significant psychomotor agitation without seizures. He is treated with oral levetiracetam, metronidazole, carnitine, and a protein-restricted diet (~1 g/kg/day). In the last evaluation, after anorexia and weight loss, without clinical decompensation or metabolic acidosis, high excretion of MMA in urine and elevated serum MMA levels persisted.

Clinical review of reported patients with MCE deficiency

To date, 22 cases of MCE deficiency have been published in English [4-14]. The available information has been reviewed and summarized in Table 3. Patients 1 and 2 are the siblings described in this case report; the other cases are ordered by publication date. Patients 4 and 5, 7 and 8, and 16 and 17 are siblings.

**Table 3:** Clinical, biochemical, and genetic characteristics of the cases with biallelic MCEE mutations MMA: Methylmalonic acid; MCA: Methylcitric acid; C3: Propionylcarnitine; Cr: Creatinine; MCEE: Methylmalonyl-CoA epimerase gene; SPR: Sepiapterin reductase; n.r.: Non -reported; n.a.: Non applicable; y.o.: Year old; mo(s): Month(s) old *Based on the patient's fibroblasts studies

Cases	Age at onset	Age at diagnosis	Metabolic acidosis	Neurological impairment	Psychomotor development	Urinary MMA (μmol/mmol Cr, r.v.< 0.3)	Urinary MCA (μmol/mmol Cr, r.v.< 7,9)	C3 (μmol/L, r.v.0,69-3,39)	Plasma Cr	MCEE Allele 1	MCEE Allele 2	SPR deficiency	Vit B12 response	Protein restriction	Reference
Patient 1	1 mo.	11 y.o.	n.r.	Coma, epilepsy, hypotonia/ spasticity	Intellectual disability	11.4-37.0	4.4-7.9	Elevated	Low	c.139C>T	c.139C>T	n.r.	No	Yes	This article
Patient 2	1 mo.	5 y.o.	Yes	Coma, epilepsy, spasticity, ataxic gait	Global delay	15.2-1230.9	126.5 mg/g Cr	12.07	Normal	c.139C>T	c.139C>T	n.r.	No	Yes	This article
Patient 3	2 y.o.	16 y.o.	No	Spasticity, ataxia, hypotonia, dystonia	Motor delay	40 - 1175	n.r.	2.7- 5.6	n.r.	c.139C>T	c.139C>T	Yes	No*	Yes	[4,6]
Patient 4	13 mo(s).	12 y.o.	Yes	No	Normal	180 - 1456	Elevated	n.r.	n.r.	c.139C>T	c.139C>T	n.r.	No*	During illness	[5]
Patient 5 (sibling of patient 4)	n.a.	14 y.o.	No	No	Normal	95-166	n.r.	n.r.	Normal	c.139C>T	c.139C>T	n.r.	n.a.	Yes, self-restricted	[5]
Patient 6	3 y.o.	n.r.	n.r.	Spasticity, ataxia, dysarthria	Motor delay	621	n.r.	n.r.	n.r.	c.178A>C	c.178A>C	n.r.	No*	n.r.	[6]
Patient 7	n.a.	n.r.	No	No	Normal	1400	n.r.	n.r.	n.r.	c.139C>T	c.139C>T	n.r.	No*	n.r.	[6]
Patient 8 (sibling of patient 7)	1 mo.	16 y.o.	No	Dystonia, oculogyric crisis, cataplexy	Global psychomotor delay	60	n.r.	n.r.	n.r.	c.139C>T	c.139C>T	Yes	No	n.r.	[8]
Patient 9	15 mo(s).	n.r.	Yes	No	Near normal	Elevated	Elevated	Elevated	n.r.	c.139C>T	c.139C>T	n.r.	n.r.	Yes	[7]
Patient 10	5 y.o.	n.r.	Yes	Transient confusion and hallucination	Normal	47 - 151	83	1.53 - 21.6	n.r.	c.139C>T	c.379-644A>G	n.r.	No*	During illness	[9]
Patient 11	5 y.o.	5 y.o.	Yes	Attentional deficit	Language delay	18 - 212	Elevated	17.5	n.r.	c.139C>T	c.139C>T	n.r.	n.r.	Yes	[10]
Patient 12	4 mo.	n.r.	n.r.	n.r.	n.r.	441	n.r.	n.r.	n.r.	c.139C>T	c.139C>T	n.r.	No*	n.r.	[11]
Patient 13	2 y.o.	n.r.	Yes	n.r.	n.r.	458	Elevated	Elevated	n.r.	c.139C>T	c.139C>T	n.r.	No*	n.r.	[11]
Patient 14	2.5 y.o.	n.r.	Yes	n.r.	n.r.	Elevated	Elevated	Elevated	n.r.	c.139C>T	c.139C>T	n.r.	No*	n.r.	[11]
Patient 15	1.5 y.o.	n.r.	Yes	n.r.	n.r.	594	n.r.	n.r.	n.r.	c.139C>T	c.139C>T	n.r.	No*	n.r.	[11]
Patient 16	n.r.	n.r.	n.r.	n.r.	n.r.	Elevated	n.r.	n.r.	n.r.	c.139C>T	c.139C>T	n.r.	No*	n.r.	[11]
Patient 17 (sibling of patient 16)	n.r.	n.r.	n.r.	n.r.	n.r.	Elevated	n.r.	n.r.	n.r.	c.139C>T	c.139C>T	n.r.	No*	n.r.	[11]
Patient 18	2 y.o.	n.r.	n.r.	Hypotonia, legs’ spasticity	Motor delay	143	n.r.	n.r.	n.r.	c.139C>T	c.139C>T	n.r.	No*	n.r.	[11]
Patient 19	1 mo.	n.r.	n.r.	Seizures	Global delay	Elevated	Elevated	Elevated	n.r.	c.139C>T	c.139C>T	n.r.	No*	n.r.	[11]
Patient 20	6 mo.	n.r.	n.r.	Seizures following viral infection	Normal	143-184	Elevated	Elevated	n.r.	c.158T>G	c.158T>G	n.r.	No*	n.r.	[11]
Patient 21	< 1 y.o.	n.r.	Yes	n.r.	n.r.	Elevated	n.r.	n.r.	n.r.	c.139C>T	c.139C>T	n.r.	No*	n.r.	[11]
Patient 22	n.r.	78 y.o.	No	Parkinson, strokes, hallucinations	n.r.	5.5-60	n.r.	13	High	c.139C>T	c.419delA	No	No	n.r.	[12]
Patient 23	7 mo.	10 mo.	No	No	Global delay	Elevated	n.r.	3.27-14.38	n.r.	c.296T>C	c.296T>C	n.r.	Yes	Yes	[13]
Patient 24	3.5 y.o.	3.5 y.o.	Yes	Confusion during gastroenteritis	Normal	0-1234	n.r.	7.7	n.r.	c.139C>T	c.139C>T	No	Not tried	During illness	[14]

Patients Homozygous for the c.139C>T (p.Arg47*) Variant (17 Cases From 14 Families)

The first patient with MCE deficiency (patient 3) was described in 2006. He harbored the c.139C>T (p.Arg47*) variant in homozygosity [4]. Sixteen more patients homozygous for that variant were recognized [4-8,10,11,14]. All presented in infancy or childhood with a moderate to high excretion of MMA in urine.

At least two patients (3 and 8) from unrelated families had sepiapterin reductase (SPR) deficiency with pathogenic variants in the *SPR* gene [4,8]. Both presented with predominant motor delay and progressive dystonia. Two more patients (18 and 19) presented with significant neurological impairment [11], one with hypotonia and spasticity and the other with seizures and global psychomotor delay. No information about the *SPR* gene was available.

Metabolic acidosis was disclosed in eight patients in this group. Patient 12 presented with an isolated cardiomyopathy [11]. Patients 5 and 7 were asymptomatic [5,6]. No clinical information was available for patients 16 and 17 [11]. A single patient had a clinical response to vitamin B12 [5]. The other patients were either unresponsive to vitamin B12 or had no information regarding vitamin B12 supplementation. Protein restriction in the diet was recorded in patients 3, 5, 9, and 11.

Patients With a c.139C>T (p.Arg47*) Variant in Compound Heterozygosity

At least two patients (10 and 22), compound heterozygous for the most common c.139C>T (p.Arg47*) variant with a different *MCEE* variant, have been described so far [9,12]. Patient 10, reported in 2016, was heterozygous for the common variant and the intronic variant c.379-644A>G [9]. He presented at five years of age with metabolic acidosis, transient confusion, and hallucinations during the episode. He had high MMA, MCA, and 3-HP levels in urine, plasma, and cerebrospinal fluid and elevated serum C3. During follow-up, a moderate elevation of urinary MMA, MCA, and serum MMA persisted. His fibroblasts were unresponsive to hydroxocobalamin. Protein restriction was prescribed only during intercurrent illnesses. At 11 years old, he had normal intellectual abilities [9].

Patient 22, reported in 2019, was 78 years old at diagnosis. He had a medical history of Parkinson’s disease, two strokes, basal ganglia infarctions in brain MRI, and cognitive deterioration with confusion and hallucinations. Elevation of serum MMA, a moderate excretion of MMA in urine, and increased plasma levels of C3 were disclosed. A 'clinical whole genome sequencing' revealed the *MCEE* variant c.419delA (p.Lys140Argfs*6) in compound heterozygosity with the common variant. He was unresponsive to hydroxocobalamin supplementation; dietary protein restriction was not mentioned [12].

Patients Homozygous for Other MCEE Variants

Patient 6, harboring the c.178A>C (p.Lys60Gln) homozygous variant in *MCEE*, presented at the age of three years old with spasticity, ataxia, dysarthria, and motor delay, without metabolic acidosis [6]. Patient 20, homozygous for the *MCEE *variant c.158T>G (p.Ile53Arg), presented with seizures following a viral infection at six months. His psychomotor development was normal. Urinary MMA, MCA, and C3 in the blood were elevated [11]. Patient 23, homozygous for the *MCEE* variant c.296T>C (p.Leu99Pro), presented at seven months with global psychomotor delay and elevation of MMA and C3. Intramuscular methylcobalamin decreased C3 and C3/C2 levels; a reduction in urinary MMA was not mentioned [13].

## Discussion

**Figure 3:**
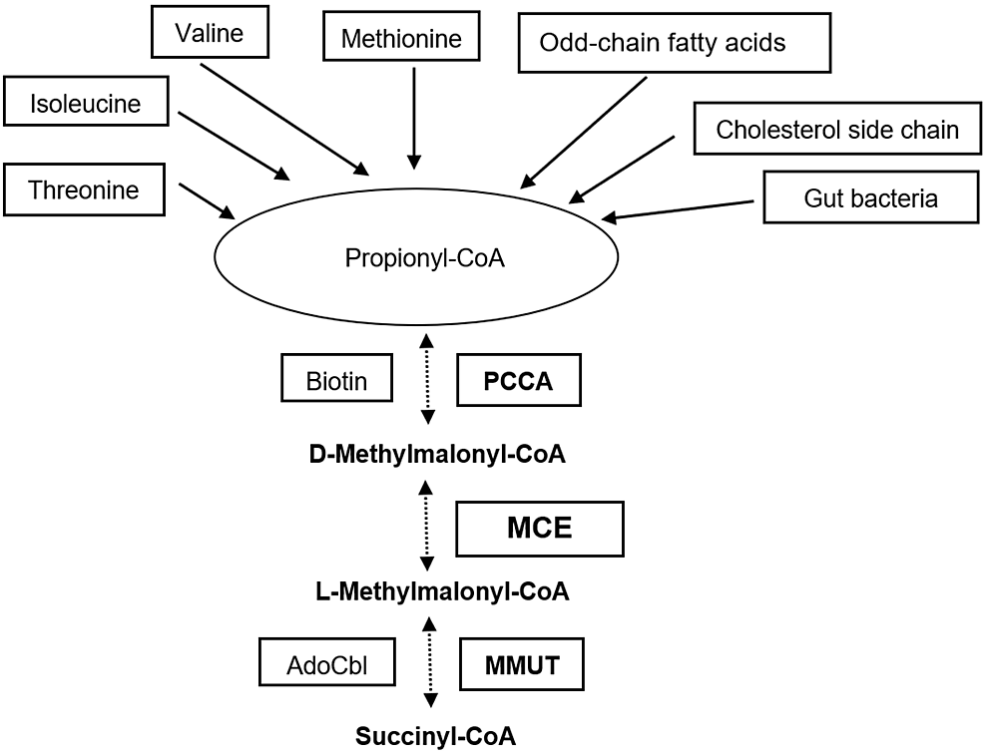
Pathway of mitochondrial propionate catabolism Biotin and AdoCbl are the co-factors of PPCA and MMUT carboxylase, respectively. There is no known co-factor for MCE. AdoCbl: Adenosylcobalamin; PPCA: Propionyl-CoA carboxylase; MMUT: Methylmalonyl-CoA mutase; MCE: Methylmalonyl-CoA epimerase; CoA: Coenzyme A

MCE deficiency is an ultra-rare disorder that should be considered in the differential diagnosis of isolated MMAuria. Raised MMA, MCA, and C3 levels due to MCE deficiency, which impairs the propionate catabolic pathway in mitochondria, are the biochemical hallmark of the disease. They also occur in MMUT and AdoCbl deficiencies, which disturb the same metabolic pathway downstream (Figure 3). Thus, genetic testing is indispensable for correct diagnosis in patients with overlapping clinical features and predominant neurological manifestations. Like MCE, AdoCbl deficiencies (cblA, cblB, and cbl-D-MMA) are rare disorders, with MMUT deficiency being the less rare and most well-studied disorder. Transient-isolated MMAuria has also been reported in a few cases with pathogenic variants in* TCblR/CD320 *[16], the gene for cellular uptake of transcobalamin-bound vitamin B12, detected in neonatal screening in some countries. We did not investigate this gene.

We performed a MEDLINE and EMBASE search on July 5, 2023, with the following search terms: ("methylmalonyl-CoA epimerase" OR "methylmalonyl-CoA racemase" OR "MCE deficiency" OR "*MCEE* gene") AND (English [Language]). Eleven of the 53 articles found included case reports of patients with MCE deficiency, for a total of 22 patients with variants in the two alleles of the *MCEE* gene [4-14]. Including the two novel patients presented here, 24 cases of MCE deficiency from 20 unrelated families have been described (in English). Methylmalonic aciduria was reported in all cases, with highly variable levels unrelated to clinical severity or genetic diagnosis. Patient 2 had less severe clinical manifestations and higher urinary and serum AMM than his elder brother (patient 1). Spot MMAuria is highly variable, and 24-hour urine collection is not accessible in young children or the neurologically impaired [17]. Urine MCA and blood/plasma C3 levels were also elevated in all the patients reported (10 patients and 13 patients, respectively), including patients 1 and 2.

Like MMUT or AdoCbl deficiencies, metabolic acidosis attacks due to the accumulation of MMA, MCA, and other organic acids, including lactate and ketone bodies, can occur in MCE-deficient patients. Excessive protein intake or catabolic periods, such as underlying infection or prolonged fasting, predispose to these events. Metabolic acidosis was reported in 10 out of 22 patients. The patients we report had several metabolic crises during febrile infections, mainly in infancy. Although we have no information on patient 1’s laboratory investigation before five years of age, we can assume he also had metabolic acidosis attacks. Hyperammonaemia, reported in patient 2 (although ammonium levels were unavailable) due to secondary impairment of the urea cycle, is a common complication in metabolic decompensation episodes of organic acidurias due to mitochondrial enzyme deficiencies, namely in MMUT deficiency. Interestingly, ammonium levels were reported only in patient 10 (107 μmol/L) and patient 23 (normal).

Renal involvement is a significant concern in MMUT deficiency due to the toxic effects of MMA and other accumulated metabolites. Plasma creatinine was documented only in two of the 22 patients previously reported (Table 3). Creatinine may not be the best parameter to evaluate renal function in cases with low muscle bulk, as we could see in patient 1. Cystatin C might help estimate the glomerular filtration rate in these patients.

Patients homozygous for the c.139C > T (p.Arg47*) mutation account for 19 of the 24 total known cases (79%), including 17 patients previously reported and the two siblings here presented. This nonsense variant creates a truncated protein and is classified as pathogenic in ClinVar. It was first described by Bikker et al. [4] in 2006 as causing mild MMAuria and was soon considered the most frequent cause of "atypical" MMAuria [6]. These classifications, i.e., "mild" and "atypical," were probably assigned by comparison with the better-known MMAuria due to MMUT deficiency. However, MMA (as other metabolites) levels are highly variable among disorders, patients, and over time in the same patient and are not related to clinical severity, as we observed in patients 1 and 2. Presentations were highly variable among patients homozygous for this nonsense mutation, ranging from asymptomatic to significant neurological impairment, acute metabolic acidosis, or cardiomyopathy, so we found no genotype-phenotype association.

The c.139C > T (p.Arg47*) variant was found in heterozygosity in two patients (10 and 22). In patient 10, the presence of this variant segregating with the (deep) intronic variant c.379-644A>G might explain the phenotype. The second variant was shown to create a new consensus donor splice site, GTAA, resulting in a premature stop codon, which suggests an impact on protein function [9]. Also, the likely pathogenic variant c.419delA (p.Lys140Argfs*6), found in patient 22, is a frameshift, introducing a premature stop codon, leading to a truncated protein [12].

Patient 6, reported in 2007, was the first reported individual with a variant in homozygosity different from c.139C > T (p.Arg47*): c.178A>C (p.Lys60Gln). This missense variant was predicted not to be deleterious by bioinformatic predictors (sorting intolerant from tolerant (SIFT) and polymorphism phenotyping (POLYPHEN)), and the structural, biophysical, and enzymatic assessments indicate that this variant does not modify protein structure and function [11]. Only two other patients with different *MCEE* variants in homozygosity have been described: patient 20 [11] and patient 23 [13], with c.158T>G (p.Ile53Arg) and c.296T>C (p.Leu99Pro) variants, respectively. The former is a missense variant predicted to be deleterious by SIFT and POLYPHEN. Moreover, the Ile-to-Arg change causes reduced thermostability and the inability to fold the protein correctly. The latter, concerning patient 23, is a missense, likely-pathogenic variant whose protein consequence is unknown.

Most patients presented with neurological impairment (12 patients) or psychomotor delay or intellectual disability (10 patients). Epilepsy and motor tone abnormalities were the most commonly pointed-out abnormalities. In five cases, there was no neurological impairment (patients 4, 5, 9, 21, and 23), and in seven, neurodevelopment was referred to as normal or near normal, although the length of follow-up was not reported. Our patients had a severe clinical presentation at an early age and were under suboptimal medical care, which probably determined the current neurological sequelae.

Clinical presentation occurred most often in infancy, namely in the first months of life, as in patients 1 and 2. Patient 22, reported in 2019, seems to be an exception, with a presentation apparently in adulthood and a diagnosis at 78 years old [12]. He had a medical history of chronic renal failure, cardiomyopathy, Parkinson’s disease, two stroke episodes, basal ganglia infarctions, and cognitive deterioration. Elevation of MMA and C3 led to the diagnosis. A single patient with MCE deficiency (patient 12) presented with an isolated cardiomyopathy [11]. Interestingly, basal ganglia stroke, chronic renal failure, and cardiomyopathy are well-known complications of MMUT and Cbl metabolism deficiencies [3]. Methylmalonic acid accumulation, although moderate, may play a role in the pathophysiology of these complications in MCE deficiency.

Patients 3 and 8, from unrelated families, had SPR deficiency with mutations in the *SPR* gene [4,8]. Most of their symptoms could be attributed to this concomitant disorder since both presented with predominant motor delay and progressive dystonia. Patient 8 had a normal brain MRI, as frequently happens in neurotransmitter disorders. Since both the *MCEE* and *SPR* genes are located on chromosome 2, a contiguous gene syndrome was ruled out in these patients [8]. Patient 8’s brother, patient 7, was asymptomatic despite sharing the homozygous *MCEE* variant and having MMAuria, reinforcing the role of SPR deficiency in patient 8's clinical phenotype. Patient 5, who, like patient 7, performed genetic testing because of his affected sibling, was also asymptomatic [5].

The brothers described here (patients 1 and 2) also have significant neurological impairment, spasticity, epilepsy, and psychomotor delay or intellectual disability. Although clinical information before their arrival in our country is scarce, we can assume that repeated metabolic crises may have caused cerebral damage, mainly in the older sibling. He was only diagnosed with MMAuria and treated as an MMUT deficiency at age five when his brother presented with a coma. Consequently, his motor function (wheelchair-dependent), cognitive performance, and brain MRI lesions grew worse than his brother’s. SPR deficiency still needs to be investigated in our patients since it was not included in the gene panel that was performed. The normal prolactin levels, although reassuring, do not exclude a tetrahydrobiopterin metabolism disorder causing a dopamine deficit. Sepiapterin reductase deficiency, which could contribute to the severity of the neurologic manifestations, should be investigated in our patients by genetic testing, and a therapeutic trial with dopamine should be considered.

Adenosylcobalamin is the cofactor of MMUT, which converts L-MMA, resulting from the epimerase action upon D-MMA, into succinyl-CoA. We stopped vitamin B12 supplementation because we did not expect it to be helpful in this disorder. Nonetheless, according to protocol, we did not evaluate the biochemical responsiveness to vitamin B12 [18]. Patient 4 was thought to have a clinical response to intramuscular hydroxocobalamin, but urinary MMA levels remained high, and her fibroblasts were unresponsive [5]. Patient 23 is the only MCE deficiency patient with a biochemical response to vitamin B12, as intramuscular methylcobalamin decreased C3 and C3/C2 levels; a reduction in MMA was not mentioned. [13]. She was homozygous for the *MCEE* variant 296T>C (p.Leu99Pro), reported as likely pathogenic by the authors, but its consequences regarding protein functionality are unknown. The apparent response to methylcobalamin would point to a cobalamin metabolism disorder. Nevertheless, no mutations were found in the *MMAA*, *MMAB*, and *MMADHC* genes. All the other patients were either unresponsive to vitamin B12 (15 patients) or had no information regarding vitamin B12 supplementation (Table 3).

Since approximately 50% of MMA originates from four amino acids, protein restriction is a therapeutic option in isolated MMAurias, mostly in vitamin B12-non-responsive MMUT deficiency [1]. Protein restriction was usual in the revised patients’ cohort (Table 3). Although its efficacy is unknown in MCE deficiency, at least during underlying illnesses, it might prevent metabolic decompensation and neurological sequelae, as stated in the current therapeutic guidelines for MMUT deficiency [15]. Non-toxic amino acid mixture supplementation in MMUT deficiency is controversial, and recent guidelines do not recommend it. Our patients do not have amino acid mixture supplementation following those guidelines. They are medicated with oral metronidazole to reduce the propionate from gut bacteria metabolism. Neither amino acid mixtures nor metronidazole were reported in other patients. Due to its consistent biochemical profile and potential for dietary and medical treatment, MCE deficiency could be included in neonatal screening programs if C3 levels are high enough in the first few days of life.

## Conclusions

Methylmalonyl CoA epimerase deficiency clinical features are primarily neurological: acute metabolic acidosis with coma, seizures, stroke, or chronic evolution with motor abnormalities and intellectual disability. Clinical severity varies, with few asymptomatic cases with genetic and biochemically confirmed disease. We found no association between genotype and biochemical and clinical phenotypes. Since MCE shares a similar biochemical profile and overlapping clinical features with other disorders of propionate catabolism, genetic testing is indispensable for diagnosis. In our patients, the correct diagnosis allowed discontinuation of vitamin B12 and adequate genetic counseling for the family.

## Appendices

Appendix A

Genes in the Genetic Metabolic Diseases Panel

ABCD4; ABCG2; ACAD9; ACADL; ACADM; ACADS; ACADSB; ACADVL; ACSF3; ACY1; AGL; AGXT; ALDH4A1; ALDH6A1; ALDOA; ALDOB; ALPL; AMT; ANO5; ARG1; ARSB; ASL; ASPA; ASS1; BCKDHA; BCKDHB; BTD; CBS; CPS1; CPT1A; CPT1B; CPT2; CTNS; CYP27A1; D2HGDH; DBT; DDC; DGUOK; DHCR7; DMGDH; DYSF; EBP; ECHS1; ENO3; ETFA; ETFB; ETFDH; ETHE1; EXT1; EXT2; FAH; FBP1; FDX1L; FH; FKRP; FKTN; FLAD1; FMO3; G6PC; G6PC3; GAA;GALE; GALK1; GALNS; GALT; GAMT; GATM; GBE1; GCDH; GCH1; GCK; GCSH; GK; GLA; GLB1; GLDC; GLUD1; GPHN; GUSB; GYG1; GYS1; GYS2; HADH; HADHA; HADHB; HCFC1; HEXA; HGSNAT; HK1; HLCS; HMGCL; HMGCS2; HPD; HSD17B10; IDUA; ISCU; IVD; KCNJ11; L2HGDH; LDHA; LIPA; LMBRD1; LPIN1; MAT1A; MCCC1; MCCC2; MLYCD; MMAA; MMAB; MMACHC; MMADHC; MOCS1; MOCS2; MPI; MTHFR; MTR; MTRR; MUT; MVK; NAGS; NPC1; NSDHL; OAT; OTC; OXCT1; PAH; PC; PCCA; PCCB; PCK1; PCK2; PFKM; PGAM2; PGK1; PGM1; PHKA1; PHKA2; PHKB; PHKG1; PHKG2; PMM2; POLG; PPARG; PRKAG2; PRODH; PYGL; PYGM; QDPR; RFK; SIL1; SLC16A1; SLC22A5; SLC25A13; SLC25A15; SLC25A20; SLC25A32; SLC2A1; SLC37A4; SLC3A1; SLC52A1; SLC52A2; SLC52A3; SLC6A8; SLC7A7; SLC7A9; SUGCT; TANGO2; TAT; TAZ; TCN2; TMEM70; TSEN54; UCP2; UMPS
